# An Overview of Laryngeal Cancer Treatment at a Tertiary Care Oncological Center in a Developing Country

**DOI:** 10.7759/cureus.2730

**Published:** 2018-06-01

**Authors:** Mohammad Adeel, Muhammad Faisal, Asma Rashid, Sadaf Usman, Usman Khaleeq, Taskheer Abbas, Abdul Rehman, Kashif Malik, Raza Hussain, Arif Jamshed

**Affiliations:** 1 Head and Neck Oncology, Shaukat Khanum Memorial Cancer Hospital and Research Center, Lahore, PAK; 2 Department of Surgical Oncology, Shaukat Khanum Memorial Cancer Hospital and Research Center, Lahore, PAK; 3 Clinical and Radiation Oncology, Shaukat Khanum Memorial Cancer Hospital and Research Center, Lahore, PAK; 4 Radiation Oncology, Shaukat Khanum Memorial Cancer Hospital and Research Center, Lahore, PAK

**Keywords:** squamous cell carcinoma, radiotherapy, chemo-radiotherapy, salvage laryngectomy, pharyngocutaneous fistula

## Abstract

Introduction

Development of laryngeal cancer is multifactorial, and management is surrounded with controversies. Recent reports suggest a decline in the survival of these patients. We conducted a study to analyze the clinicopathological parameters and compute the outcomes in terms of survival in patients with laryngeal cancer treated at our institution.

Methods

Electronic charts of 515 patients with Laryngeal cancer treated at our Hospital and Research Center from 2004 to 2014 were retrospectively reviewed.

Results

Median age was 62 years. Male: female ratio 91%: 9%. Sixty-two percent were smokers. Histologically, all were squamous cell carcinoma. Most common subsite was glottis (88%). Treatment was non-surgical in 92% and surgical in 8%. The five-year overall survival (OS), disease-specific survival (DSS), disease-free survival (DFS) and locoregional control (LRC) were 67%, 74%, 59% and 70%, respectively. OS, DSS, DFS and LRC for early stage (I-II) and advance stage (III-IV) were 81 and 54%, 86 and 63%, 75 and 45%, and 83 and 57%, respectively. Twenty-two percent recurred locally. Of these failures, 19% were inoperable, 36% were surgically salvaged and 34% refused laryngectomy.

Conclusions

Our survival rates are comparable with published data. The high refusal rate for salvage total laryngectomy is concerning and needs further study to evaluate the reasons.

## Introduction

Squamous cell carcinoma (SCCA) of larynx is amongst the most common head and neck cancers that account for about 2.4% of newly diagnosed cases and 0.7% of all cancer-related deaths occurring worldwide/year [1]. The incidence and mortality is lower for women as compared to men [2]. The development of laryngeal cancer is multifactorial. Smoking and alcohol consumption are the most common risk factors especially in developed countries [3]. Five-year overall survival of laryngeal cancers has been reported from 32 to 70% cases [4].

According to national cancer database reports, the survival of patients with SCCA of larynx is on decline in the United States and many attribute it to the increasing use of organ preservation management plans for the advance laryngeal cancers [5,6]. Management of laryngeal SCCA has always been a topic of debate but there is no argument on the fact that its treatment is multidisciplinary. For early laryngeal tumors (T, T2), transoral surgery with or without laser or radiotherapy alone has shown comparable results [7], whereas surgery followed by radiotherapy is considered for advanced cancers [8]. More recently, concurrent chemoradiotherapy is replacing surgery as a treatment modality for advanced laryngeal cancers with almost similar outcomes [9,10]. Even though non-surgical management is widely replacing surgery, total laryngectomy still has an important role in advanced and recurrent disease.

The aim of this study was to retrospectively analyze the treatment outcomes of laryngeal cancers treated at a tertiary care cancer center in a developing country.

## Materials and methods

Our Head and Neck unit prospectively maintains a detailed database on all patients with head and neck cancer treated at a tertiary care hospital. We retrospectively reviewed the clinicopathologic data of all laryngeal cancer patients, who were treated at our institution from January 2004 to December 2014. Our database identified a total of 652 cases with biopsy-proven laryngeal cancer in the given period. The period was chosen to ensure a minimum follow-up of two years. Eighty-nine patients consulted our clinic only for opinion and did not seek treatment in our center. Twenty-seven patients had palliative treatment because of their advanced stages, whereas, nine cases absconded their treatment and twelve had non-squamous cell histology, hence all these patients were excluded, and data was collected of remaining 515 cases having squamous cell carcinoma who underwent a radical treatment.

All tumors were staged according to the AJCC 7th edition (American Joint Commission on Cancer). Our institutional practice is to recommend concomitant chemoradiotherapy (CRT) in patients with locally advanced laryngeal cancer except those with compromised airways or extensive cartilage involvement. Patients who refuse primary laryngectomy are also treated with concomitant CRT.

We used Statistical package for social sciences, version 20 for statistical analysis. Locoregional control (LRC) was our primary endpoint with secondary endpoints being overall survival (OS), disease-specific survival (DSS) and disease-free survival (DFS). Patients terminally ill at last follow-up were considered dead. Deaths and lost to follow-up were considered as events for overall OS and DFS.

LRC was defined as the time interval from the date of start of treatment until date of loco-regional failure or censored at the date of death if the patient died from noncancerous reason but without relapse, or date last seen if alive and relapse-free. OS was calculated from starting date of treatment till the death date for those who died either because of disease or due to non-cancer causes or censored at the last follow-up date seen alive. DSS was calculated until death date if the patient died from laryngeal carcinoma, otherwise censored at the date of death due to noncancerous reasons or alive on last follow-up date. DFS was calculated from the start of treatment to the date of recurrence and death due to laryngeal cancer. Survival curves were obtained according to Kaplan-Meier method and 95% confidence intervals for survival estimates were calculated. Survival analyses were carried out through univariate and multivariate methods. The former was primarily used to screen through the potential prognostic factors searching for any, that was significantly related to survival. The log-rank test (Mantel-Cox) was used to measure the significance. Cox Proportional Hazards Model (Forward Conditional) was applied to all significant variables (on univariate analysis) to see if the variables had an independent effect on survival.

An exemption was taken from ethical review board for this study as it was a retrospective chart review and direct contact with patients was not involved.

## Results

Our study cohort included 515 patients (470 men and 45 women; median age, 62.60 years). All of them had biopsy-proven squamous cell carcinoma of the larynx. The stage categorization was as follow: Stage I, 204 (40%), Stage II, 38 (7%), Stage III, 129 (25%), Stage IV, 133 (26%), other, 11 (2%) shown in Table 1 and Table 2.

**Table 1:** Clinicopathologic features of all patients (n = 515).

Characteristics	Number (n)	Percent (%)
Gender		
Male	470	91.3
Female	45	8.7
Smoking		
Yes	308	59.8
No	207	40.2
Smokeless tobacco		
Yes	93	18.05
No	422	81.9
Alcohol		
Yes	13	2.5
No	502	97.5
Subsite		
Supraglottis	56	10.9
Glottis	452	87.8
Subglottis	7	1.4
Histology		
Squamous cell carcinoma	515	100
Grade		
Well	193	37.5
Moderate	219	42.5
Poor	43	8.3
Unknown	60	11.7

**Table 2:** Staging and treatment. AJCC: American Joint Commission on Cancer

AJCC staging (TNM)	Number (n)	Percent (%)
T1	205	39.5
T2	43	8.3
T3	129	25
T4	127	24.7
Tis	7	1.4
TX	4	0.8
N0	474	92
N1	21	4.1
N2	20	3.9
Stage 0	7	1.4
Stage I	204	39.6
Stage II	38	7.4
Stage III	129	25
Stage IV	133	25.8
Stage x	4	0.8
Treatment		
Radiotherapy	264	51.3
Chemoradiotherapy	107	20.8
Induction chemotherapy followed by chemoradiotherapy	102	19.8
Surgery	2	0.4
Surgery, adjuvant radiotherapy	35	6.8
Surgery, adjuvant chemoradiotherapy	5	1

All patients were treated with curative intent. Radiotherapy alone was given in 264 patients (51%), 107 (30%) patients received concurrent CRT, whereas, induction chemo followed by chemoradiotherapy (C CRT) was given in 102 (20%) cases. Only 42 (8%) cases were treated with upfront surgery followed by adjuvant treatment. The pattern of recurrence is depicted in Table 3. Local recurrence was seen in 115 (22%) patients. While regional and locoregional recurrence was seen in 22 (4%) patients, distant metastasis was reported in nine (1.7%) patients. Incidence of second primary malignancy (SPM) was 3% (17 cases) and lung was the most common site of SPM.

**Table 3:** Recurrences and second primary malignancies.

Mean follow-up: 3.8 years		
Characteristics	Number (n)	Percent (%)
Site of recurrence		
Local	115	22.3
Regional	12	2.3
Locoregional	10	1.9
Distant	9	1.7
Incidence of second primary malignancy	17	3.3
Lung cancer	10	
GI cancer	2	
GU cancer	3	
Others	1 Lymphoma, 1 Sarcoma	

All patients with recurrence (n = 137) were confirmed with direct laryngoscopy and biopsy in cases of local recurrence whereas, fine needle aspiration cytology was used to confirm nodal recurrence. All cases were restaged with computed tomography (CT) scan or positron emission tomography (PET) CT where in doubt. Eight recurrences occurred in the patients who had primary surgery and were salvaged with radiotherapy, whereas, 129 patients failed non-surgical treatment. Fifty-two (38%) patients underwent salvage total laryngectomy. Forty-six (34%) patients refused salvage laryngectomy and 26 (19%) were found inoperable (Table 4).

**Table 4:** Salvage in locoregional failures (n = 129).

Salvage surgery	n	%
Total laryngectomy	52	38
Neck dissection	5	4
Refused surgery	46	34
Inoperable	26	19

Thirty patients had their salvage laryngectomy from our institution and remaining 22 patients got operated outside our hospital, however, they kept following in our combined head and neck clinic postoperatively. Previously patients were referred outside the hospital when laryngectomy services were not available in-house. Table 5 shows clinicopathologic details of patients who underwent salvage laryngectomy, of which five cases had no pathological disease but underwent surgery because of non-functional larynx. Majority of patients (17) were pathological T4 after surgery. Pharyngocutaneous fistula (PCF) was seen in 30% of cases and was mostly managed conservatively. More than half (56.6%) of our salvage laryngectomy patients were alive without disease on their last follow-up (Table 5).

**Table 5:** Clinicopathologic details of post-laryngectomy patients. PCF: Pharyngocutaneous fistula

Characteristic	n	%
Surgical margins		
No disease	5	16.6
Involved	1	3.3
1-<5 mm	10	33.3
5-<10 mm	7	23.3
>10 mm	7	23.3
Perineural invasion		
Yes	18	60
No	7	23.3
Cartilage involvement		
Yes	16	53.3
No	14	46.6
Complications		
Pharyngocutaneous fistula	9	30
Local recurrence	6	20
Regional recurrence	1	3.33
Closure of PCF		
Conservative	8	
<2 weeks	0	
2-4 weeks	3	
>1 month	5	
Flap	1	
Post-salvage laryngectomy status		
Alive	17	56.6
Alive with disease	6	20
Died	7	23.3

**Figure 1:**
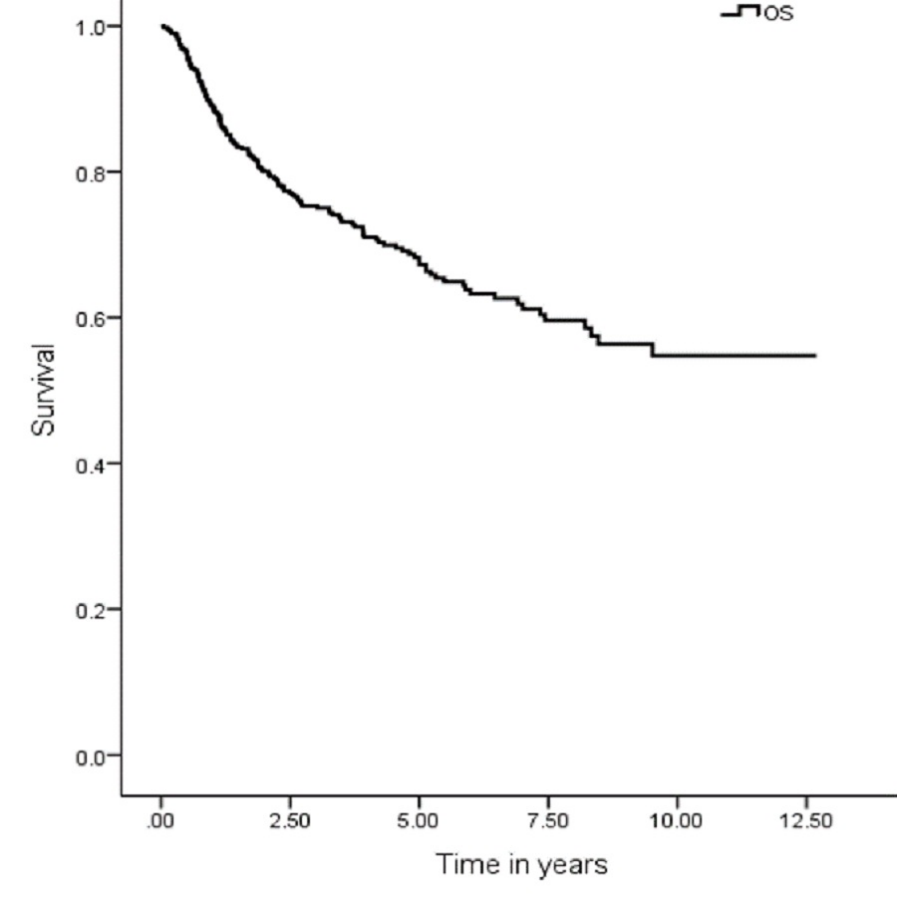
Overall survival.

**Figure 2:**
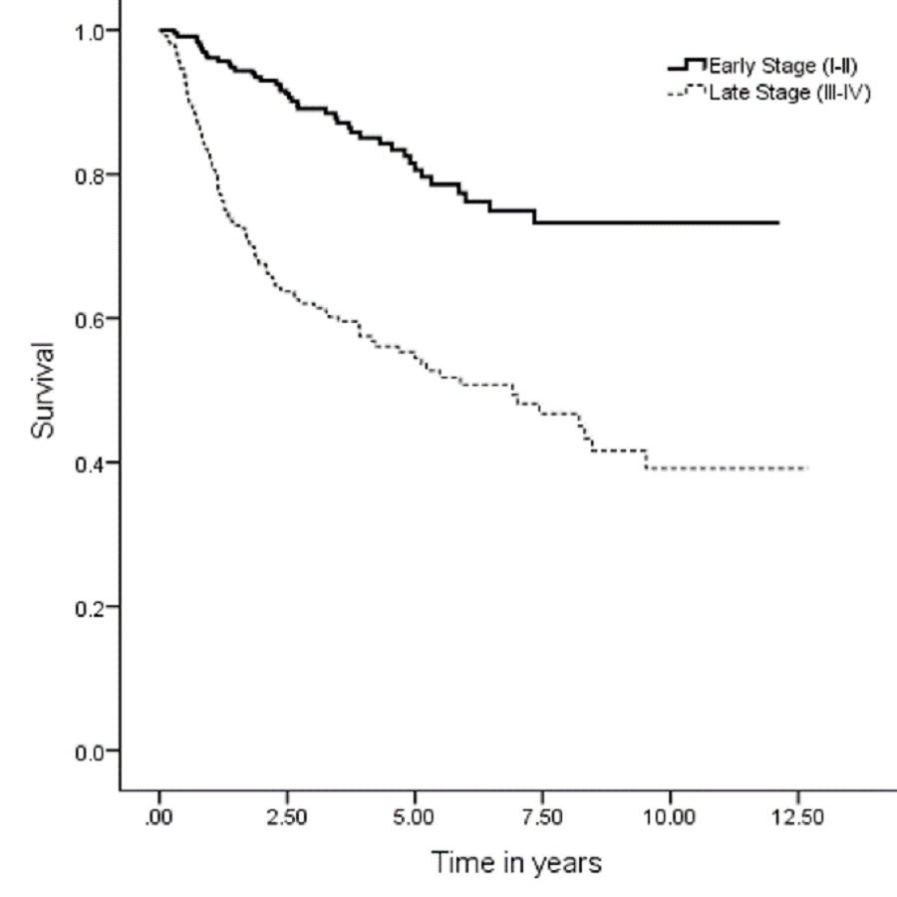
Overall survival for early and advanced cancer.

**Figure 3:**
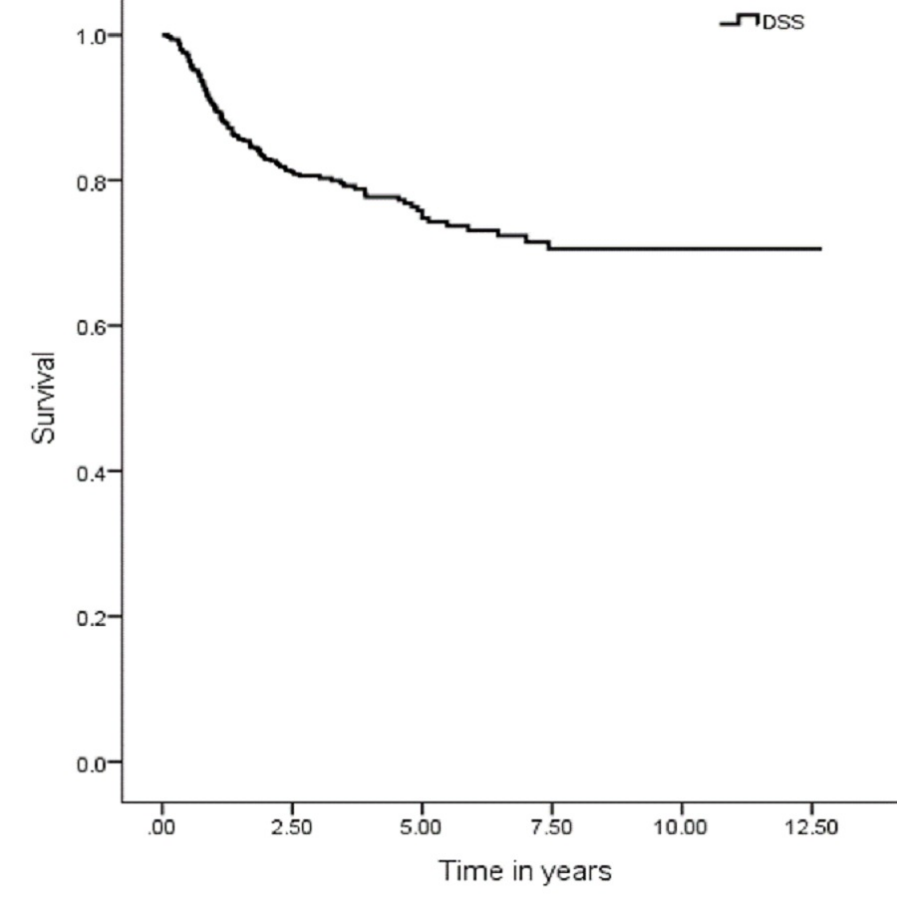
Disease-specific survival.

**Figure 4:**
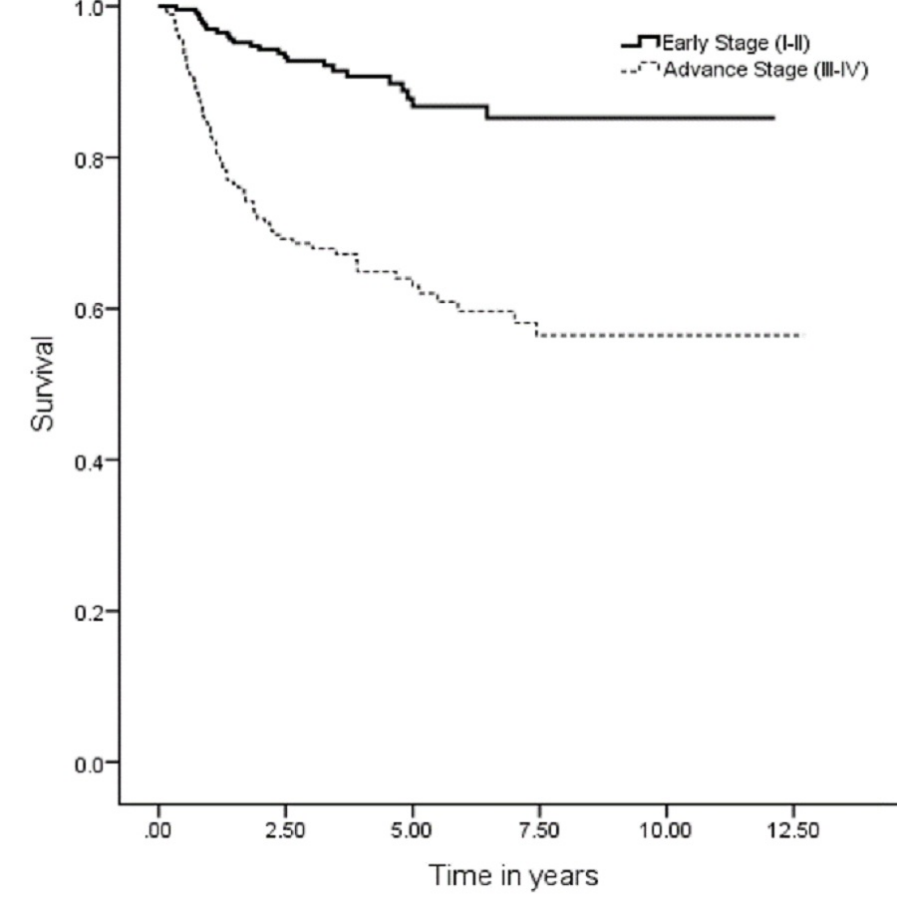
Disease-specific survival for early and advanced cancer.

**Figure 5:**
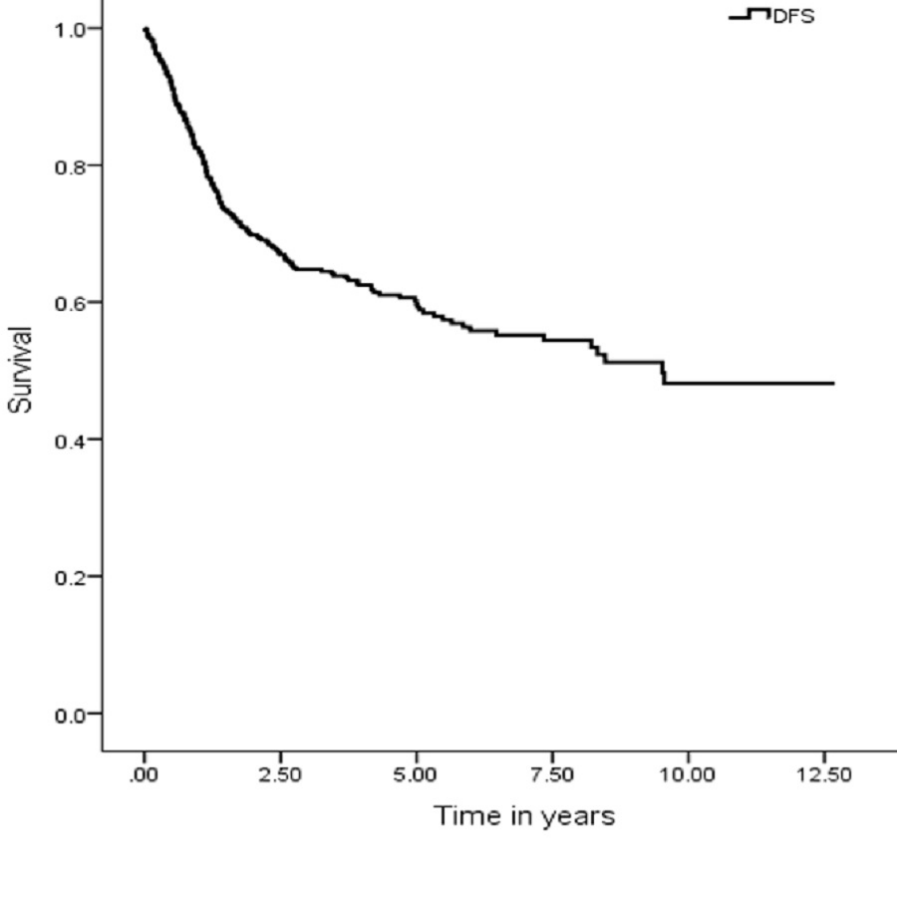
Disease-free survival.

**Figure 6:**
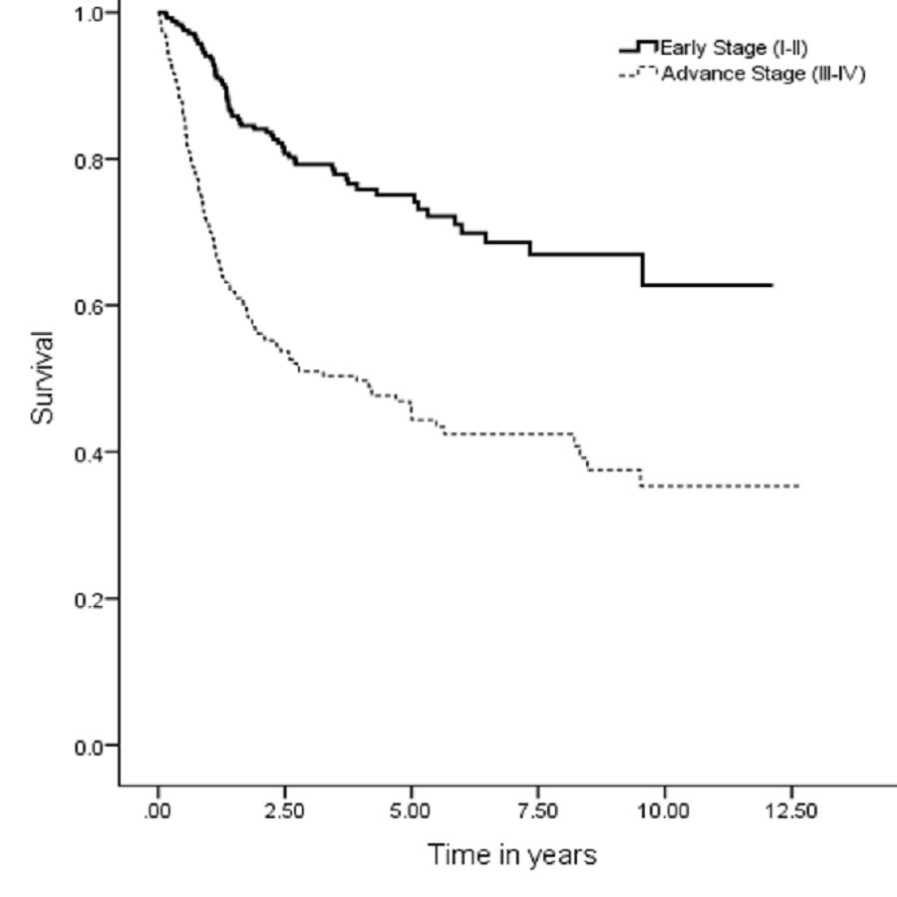
Disease-free survival for early and advanced cancer.

**Figure 7:**
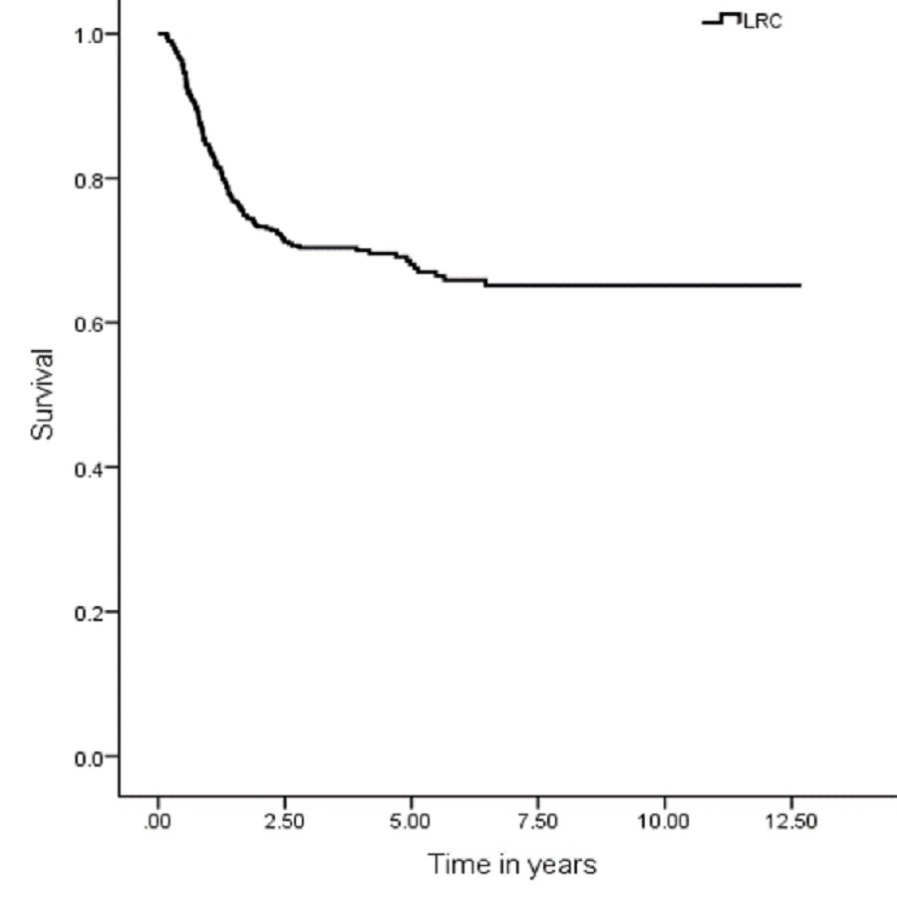
Locoregional control rate.

**Figure 8:**
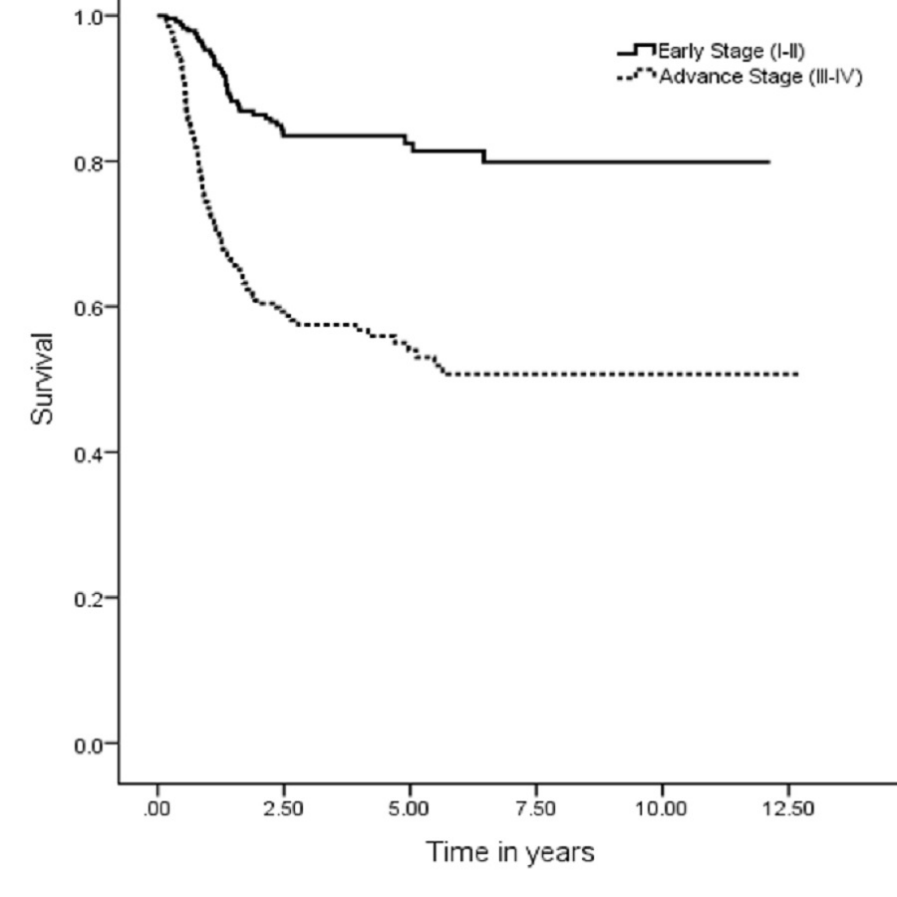
Locoregional control rate for early and advanced cancer.

The five years OS, DSS, DFS and LRC were 67 (95% CI: 7.9–9.03), 74 (95% CI: 9.1–10.1), 59 (95% CI: 9.1–10.1) and 70% (95% CI: 8.3–9.3), respectively (Figures 1, 3, 5, 7). Survival curves were also plotted for early (stage I-II) and late (stage III-IV) diseases. OS, DSS, DFS and LRC for early stage (I-II) and advance stage (III-IV) were 81 (95% CI: 9.2–10.4) and 54% (95% CI: 6.1–7.6), 86 (95% CI: 10.2–11.3) and 63 (95% CI: 7.3–8.9%), 75 (95% CI: 10.2–11.3) and 45% (95% CI: 7.3–8.9) and 83 (95% CI: 9.5–10.6) and 57% (95% CI: 6.4–7.9), respectively (Figures 2, 4, 6, 8).

Univariate analysis shown in Table 6 demonstrated that ethnicity, subsite (glottic vs supraglottis), T1 vs T2, T3 vs T4, N+ vs N- and early vs advance disease all had statistical significance in terms of survival. Multivariate analysis (Table 7) of significant variables showed that neck nodal status and stage of disease (early vs advance) were the only factors that have an independent effect on survival.

**Table 6:** Univariate analysis. Log rank (Mantel-Cox). OS: Overall survival; DSS: Disease-specific survival; DFS: Disease-free survival; LRC: Locoregional control.

Variables		5 yrs OS	p-value	5 yrs DSS	p-value	5 yrs DFS	p-value	5 yrs LRC	p-value
Gender	Male Female	68% 59%	0.991	75% 63%	0.564	59% 57%	0.438	69% 65%	0.807
Age	<50 >50	72% 66%	0.138	75% 74%	0.381	57% 59%	0.742	60% 71%	0.147
Ethnicity	Western Southern	64% 74%	0.040	71% 82%	0.044	59% 58%	0.633	70% 66%	0.976
Smokeless tobacco	Yes No	66% 68%	0.936	75% 73%	0.681	59% 61%	0.826	70% 69%	0.995
Non-surgical RX	CRT C CRT	56% 50%	0.338	62% 59%	0.931	48% 42%	0.170	55% 53%	0.470
Surgery vs non-surgery	Surgery Non-surgery	58% 66%	0.219	75% 74%	0.683	50% 59%	0.441	69% 71%	0.615
Glottic vs supraglottis	Glottis Supraglottis	70% 39%	0.000	79% 42%	0.000	62% 38%	0.000	73% 46%	0.000
T1 vs T2	T1 T2	84% 60%	0.009	90% 62%	0.000	78% 56%	0.026	85% 68%	0.012
T3 vs T4	T3 T4	65% 44%	0.000	73% 53%	0.000	54% 35%	0.003	63% 50%	0.022
N+ vs N-	N+ N-	43% 69%	0.000	45% 77%	0.000	32% 62%	0.000	39% 72%	0.000
Early vs advance	Stage I-II StageIII-IV	80% 54%	0.000	86% 63%	0.000	75% 45%	0.000	83% 57%	0.000

**Table 7:** Multivariate analysis. Cox regression model (forward conditional).

Variables		p-value
Ethnicity	Western Southern	0.44
N+ vs N-	N+ N-	0.041
Early vs Advance	Stage I-II Stage III-IV	0.000

## Discussion

Our hospital is one of its kind in the country that offers free cancer treatment to 70% of patients as it is a philanthropic institution. All patients with head and neck cancers including laryngeal cancers must go to the walk-in clinic first, where they are assessed according to the selection criterion of the hospital. Our hospital mostly does not accept advanced bulky diseases with palpable nodes hence our numbers of up front total laryngectomy are low. We lack surgical expertise in endoscopic laryngeal surgeries for early stage diseases, hence all our patients initially undergo non-surgical treatment and in case of treatment failure, salvage laryngectomy is offered.

The larynx plays an essential role in speech and communication hence organ preservation strategies in the treatment of laryngeal cancers are vital. Following the success of Veteran Affairs Laryngeal Cancer Group trial in 1990s completely turned the dynamics of laryngeal cancer management in favor of organ preservation. In our study larynx was preserved in 72% cases in the non-surgical arm which is comparable with other reports [9,11].

The median age of patients in our series was 62.6 years and male predominance of 9:1 in the epidemiology of this disease was comparable with the literature [12] and is probably attributed to the frequent addiction habits amongst males [13]. Most of our cases were glottic carcinomas (86.8%). This number was higher than other reported studies i.e., 70% [14]. This difference was probably because of the policy set up by the hospital to accept early-stage diseases and since glottic cancer is diagnosed early in its course hence the increased figures. On the other hand, subglottic cancer constituted only 1.4% of all cases consistent with other published series [15].

Almost all our stage I and II tumors were treated with radical radiotherapy. The median follow-up of our patients was 3.8 years. The five years OS, DSS, DFS and LRC in our study were 81, 86, 75 and 83%, respectively, whereas data from some of larger studies showed a figure of 83, 96, 76.7 and 82 to 87%, respectively [14,16-18].

On the other hand, in advance cases (stage III-IV) the five years OS, DSS, DFS and LRC were 54, 63, 45 and 57%, respectively, as compared to 54, 64, 30 and 65%, respectively [9,14,19]. In the present study, multivariate analysis found that nodal involvement independently affected survival which was in accordance with the literature. Various studies have reported worst prognosis in case of positive nodal status [20,21].

Our salvage rate in patients with recurrences was unsatisfactory. This was partly because of advanced locoregional disease which would render them inoperable but mostly because of the high refusal rate for salvage total laryngectomy. The refusal rate in our series was 34% which is a significant number considering surgery is now the only chance to get disease free. There is hardly any data addressing the issue of salvage total laryngectomy refusal in developing countries, but the numbers are likely to be high because of lack of education, poverty, social stigma of loss of voice, permanent tracheostomy and varying spiritual believes that leads them to seek non-medical treatment. Therefore, many operable diseases progress to inoperable. Even though, with the introduction of tracheoesophageal voice prosthesis in developed countries the issue of voice and quality of life, both have shown improvement [22]. However, the cost of this prosthesis in developing countries is still a big hurdle in its frequent use.

Although our selection of patients for salvage laryngectomy was very meticulous, disease recurred in around 43.3% patients which was on the higher side in relation to the literature, showing results between 21 and 43.2% [23-25]. This suggests the aggressive nature of these select recurrent tumors after radiation or chemoradiation. On the same note, since salvage treatment is the only option for cure, physicians and patients will always be tempted to perform salvage laryngectomy to avail that 50% chance of survival.

Salvage surgery after non-surgical treatment is known to have higher complication rate than primary surgery with an incidence of pharyngocutaneous ﬁstula up to 50% [26]. Nowadays, use of upfront myocutaneous flap is favored for salvage laryngectomy to reduce the incidence of pharyngocutaneous fistula and improve swallowing issues [27-29]. However, due to limited resources and time constraints, we do not offer upfront reconstruction in salvage patients. Still, our PCF incidence was 30% which was comparable with international literature.

Radiation-associated side effects have reduced considerably with the use of intensity-modulated radiation therapy (IMRT) because of limited dose exposure to the surrounding tissues leading to sparing of salivary glands hence reduction in xerostomia and dysphagia. Non-surgical treatment for laryngeal cancer is still associated with severe toxicity, leading to swallowing issues, speech difficulties, and a dysfunctional larynx. Unfortunately, because of the retrospective nature of the study, we were not able to retrieve reliable data on these aspects. The major strength of our study is that it is one of the biggest series reported from a developing country.

## Conclusions

Our survival rates are comparable with published data. The high refusal rate for salvage total laryngectomy is of concern and needs further study to evaluate the reasons for refusal.
